# Development and usability testing of an electronic patient-reported outcome (ePRO) solution for patients with inflammatory diseases in an Advanced Therapy Medicinal Product (ATMP) basket trial

**DOI:** 10.1186/s41687-023-00634-3

**Published:** 2023-10-09

**Authors:** Christel McMullan, Ameeta Retzer, Sarah E. Hughes, Olalekan Lee Aiyegbusi, Camilla Bathurst, Alan Boyd, Jamie Coleman, Elin Haf Davies, Alastair K. Denniston, Helen Dunster, Chris Frost, Rosie Harding, Amanda Hunn, Derek Kyte, Rebecca Malpass, Gary McNamara, Sandra Mitchell, Saloni Mittal, Philip N. Newsome, Gary Price, Anna Rowe, Wilma van Reil, Anita Walker, Roger Wilson, Melanie Calvert

**Affiliations:** 1 Centre for Patient Reported Outcome Research (CPROR), Institute of Applied Health Research, University of Birmingham, Edgbaston, Birmingham, B15 2TT UK; 2 NIHR Surgical Reconstruction and Microbiology Research Centre, University of Birmingham, Birmingham, UK; 3 Centre for Trauma Science Research, University of Birmingham, Birmingham, UK; 4 Birmingham Health Partners Centre for Regulatory Science and Innovation, University of Birmingham, Birmingham, UK; 5 National Institute for Health Research (NIHR) Applied Research Collaboration (ARC) West Midlands, Birmingham, UK; 6 National Institute for Health Research (NIHR) Biomedical Research Centre at University Hospitals Birmingham NHS Foundation Trust and the University of Birmingham, Birmingham, UK; 7 Cancer Research UK Clinical Trials Unit, University of Birmingham, Birmingham, UK; 8 Alan Boyd Consultancy, Crewe, UK; 9 Institute of Clinical Sciences, College of Medical and Dental Sciences, University of Birmingham, Birmingham, UK; 10 Aparito Ltd, Wrexham, Wales; 11 DEMAND Hub, University of Birmingham, Birmingham, UK; 12 Health Data Research UK, London, UK; 13 Academic Unit of Ophthalmology, Institute of Inflammation and Ageing, University of Birmingham, Birmingham, UK; 14 University Hospitals Birmingham NHS Foundation Trust, Birmingham, UK; 15 National Institute of Health Research Biomedical Research Centre for Ophthalmology, Moorfields Eye Hospital NHS Foundation Trust and University College London, Institute of Ophthalmology, London, UK; 16 Univeristy of Birmingham Enterprise, Birmingham, UK; 17 Birmingham Law School, University of Birmingham, Birmingham, UK; 18 Independent Adviser, Manchester, UK; 19 School of Allied Health & Community, University of Worcester, Worcester, UK; 20 Cognitant, London, UK; 21 National Cancer Institute, Bethesda, USA; 22 University Hospital Birmingham, Birmingham, UK; 23 Institute of Immunology and Immunotherapy, University of Birmingham, Birmingham, UK; 24 Research Governance, University Hospital Birmingham, Birmingham, UK; 25 National Cancer Research Institute (NCRI) Consumer Forum, London, UK; 26 Midlands Health Data Research UK, Birmingham, UK

**Keywords:** Electronic patient reported outcomes, Usability testing, Inflammatory conditions, Cognitive interviews, Early phase advanced therapy trial

## Abstract

**Background:**

Electronic patient-reported outcome (ePRO) systems are increasingly used in clinical trials to provide evidence of efficacy and tolerability of treatment from the patient perspective. The aim of this study is twofold: (1) to describe how we developed an electronic platform for patients to report their symptoms, and (2) to develop and undertake usability testing of an ePRO solution for use in a study of cell therapy seeking to provide early evidence of efficacy and tolerability of treatment and test the feasibility of the system for use in later phase studies.

**Methods:**

An ePRO system was designed to be used in a single arm, multi-centre, phase II basket trial investigating the safety and activity of the use of ORBCEL-C™ in the treatment of patients with inflammatory conditions. ORBCEL-C™ is an enriched Mesenchymal Stromal Cells product isolated from human umbilical cord tissue using CD362+ cell selection. Usability testing sessions were conducted using cognitive interviews and the ‘Think Aloud’ method with patient advisory group members and Research Nurses to assess the usability of the system.

**Results:**

Nine patient partners and seven research nurses took part in one usability testing session. Measures of fatigue and health-related quality of life, the PRO-CTCAE™ and FACT-GP5 global tolerability question were included in the ePRO system. Alert notifications to the clinical team were triggered by PRO-CTCAE™ and FACT-GP5 scores. Patient participants liked the simplicity and responsiveness of the patient-facing app. Two patients were unable to complete the testing session, due to technical issues. Research Nurses suggested minor modifications to improve functionality and the layout of the clinician dashboard and the training materials.

**Conclusion:**

By testing the effectiveness, efficiency, and satisfaction of our novel ePRO system (PROmics^R^), we learnt that most people with an inflammatory condition found it easy to report their symptoms using an app on their own device. Their experiences using the PROmics^R^ ePRO system within a trial environment will be further explored in our upcoming feasibility testing. Research nurses were also positive and found the clinical dashboard easy-to-use. Using ePROs in early phase trials is important in order to provide evidence of therapeutic responses and tolerability, increase the evidence based, and inform methodology development.

*Trial registration*: ISRCTN, ISRCTN80103507. Registered 01 April 2022, https://www.isrctn.com/ISRCTN80103507

**Supplementary Information:**

The online version contains supplementary material available at 10.1186/s41687-023-00634-3.

## Abstract (plain language summary)

More and more patients tell clinicians how they feel by completing questionnaires electronically. Therefore, it is important to assess how easy it is for patients to do this. In this study, we describe how we developed an electronic platform for patients to report their symptoms and how we tested the usability of this platform with patient partners and research nurses. Once the electronic platform was developed, quality of life and symptoms questionnaires were programmed onto it. Alerts were sent to the clinical team if specific scores were obtained on the symptoms questionnaires. Although two patient partners were not able to finish the testing session because of technical issues, the ones who completed the session liked its simplicity and responsiveness. The research nurses also liked the system and only suggested minor modifications. Following this testing, we refined the electronic platform to test it further in a larger study which investigates the safety and use of a drug. We hope that thanks to this electronic platform, we will obtain useful information on the safety and efficacy of treatment.

**Supplementary Information:**

The online version contains supplementary material available at 10.1186/s41687-023-00634-3.

## Introduction

### Background

Cell or gene therapies show great potential in treating patients with inflammatory conditions that are not responding to current treatments [1, 2]. The use and evaluation of these novel therapies, also known as Advanced Therapy Medicinal Products (ATMPs), in clinical trials, require assessment of their impact upon patients’ symptoms and health-related quality of life (HRQoL). These impacts can be assessed using patient-reported outcomes (PROs). Assessments are required at multiple timepoints, including before and at the point of receiving the therapy and over the duration of the trial. The European Medicines Agency draft guideline on safety and efficacy follow-up and risk management of ATMPs [3] states that 'Advanced Therapy Medicinal Products… because of their novelty, complexity and technical specificity may cause new risks to patients'.

A PRO is defined as “any report of the status of a patient’s health condition that comes directly from the patient, without interpretation… by a clinical or anyone else” [4]. They are collected using self-reported questionnaires, known as PRO measures. These are standardised instruments designed to capture PRO information [5]. PROs can provide evidence on the safety and efficacy of ATMPs to support marketing authorisation and provide insight into longer-term effects on patients. In addition, the tolerability of a medicinal product (the degree to which symptomatic and non-symptomatic adverse events (AEs) associated with the product’s administration), can affect the ability or desire of the patient to adhere to the dose or intensity of therapy. A complete understanding of tolerability should include direct measurement from the patient on how they are feeling and functioning while receiving treatment. It has been proposed that ‘suitable PRO tools are selected to capture patient derived data concerning the impact of the adverse events of the therapy and the overall treatment burden for the patient’ [6, 7].

PRO data were traditionally collected using paper questionnaires; however, the use of electronic PRO systems (ePRO) is becoming increasingly common [8, 9]. The equivalence of electronic and paper administration of PRO measures has also been demonstrated [10]. The use of an ePRO system can promote collection of complete PRO data from which conclusions on efficacy can be drawn. Research has shown that there are inconsistencies in how paper-based PROMs are administered by research staff during trials, which may reduce the quality of the resulting data and introduce potential bias [11]. In clinical trials where PRO best practices are not upheld, publication of PRO data has been found to be undermined [12]. Use of ePRO systems may address some of these issues through minimised administrative burden, both for patients and for trial staff; increased acceptance rates; limited secondary data entry error; and lower levels of missing data [8, 9, 13]. It has been established that use of an electronic PRO system can promote patient adherence with PRO collection in comparison to paper questionnaires [14–16]. Despite this, ePROs can also exacerbate health disparities in populations with little or no internet access.

Although ePRO systems for use in clinical trials are currently available, evidence suggests there is an additional need for such systems to accommodate “PRO alerts” [17]. PRO alerts are signals that are generated by an ePRO system and sent to the trial clinical team when a patient reports psychological or physical symptoms that require an immediate response [18]. These can be monitored and reviewed during the trial to allow timely and appropriate intervention to promote the wellbeing and safety of the patient. A system with such a feature would facilitate the systematic recording and reporting of PRO alert data, minimising co-intervention bias, promoting patient safety, and reducing liability. Such data can also be used to trigger clinician adverse event reporting. It is worth noting that PRO alerts are being increasingly used in clinical care [19]. As a result, clinical staff have become more familiar with it recently and it has been found that in a clinical setting, symptom monitoring based upon patients’ self-reports has been found to have clinical benefits [20].

Usability testing is a crucial stage of PRO development [21, 22]. It is defined by the International Organisation for Standardization (ISO) as “the extent to which a product can be used by specified user to achieve specified goals with effectiveness, efficiency, and satisfaction in a specified context of use.” [23] Usability testing ensures that the intended goals are achieved, with minimal use of resources, while promoting satisfaction of users, which is particularly important when users have a choice as to whether or not to use the product [23]. In the case of patients’ completion of PRO measures on an ePRO platform, where patient burden has been established to be a reason for non-completion and missing data [24] despite patients’ interest in these data [25], usability is particularly important. As such, when assessing the usability of the ePRO system, the context of use and characteristics of those using the ePRO system must be considered [22, 26, 27].

### Research context: POLARISE trial

This usability study is part of the PROmics^R^ study which is itself set into the context of the POLARISE Trial (ISRCTN 80103507). POLARISE (the name refers to cell polarisation) is a single arm, multi-centre, phase II basket trial investigating the safety and activity of the use of ORBCEL-C™ in the treatment of patients with Primary Sclerosing Cholangitis (PSC), Rheumatoid Arthritis (RA), Lupus Nephritis (LN) and Crohn’s Disease (CD). The aim of the POLARISE trial is to find out whether patients with primary sclerosing cholangitis (PSC), rheumatoid arthritis (RA), lupus nephritis (LN) or Crohn's disease (CD) can potentially be treated safely with a cell therapy called ORBCEL-C™ [28].

ORBCEL-C™ is a Mesenchymal Stromal Cells enriched MSC product isolated from human UCT (umbilical cord tissue) using CD362 + cell selection and was developed by Orbsen Therapeutics Ltd. in conjunction with NHS Blood and Transplant (NHSBT).

### Aims

The aim of this study is twofold: (1) to describe how we developed an electronic platform for patients to report their symptoms, and (2) to develop and undertake usability testing of an ePRO solution for use in a study of cell therapy which seeks to provide early evidence of efficacy and tolerability of treatment and test the feasibility of the system for use in later phase studies. Usability testing was undertaken to refine the system ready for use within an ATMP clinical trial, in addition to finalising training, instructional, and consent materials.

## Methods

### Ethical considerations

Usability testing was approved by the University of Birmingham Research Ethics committee (ERN_19-1271).

### Development of the PROmics^R^ ePRO system

#### Atom5™

**Fig. 1:**
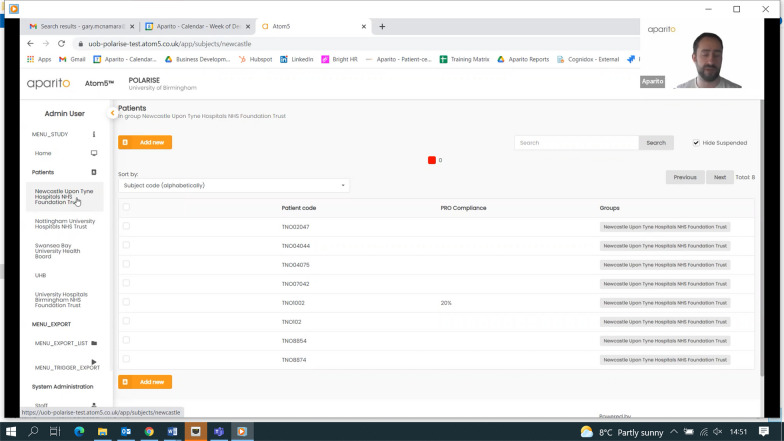
Screenshot of Atom5™ dashboard

**Fig. 2:**
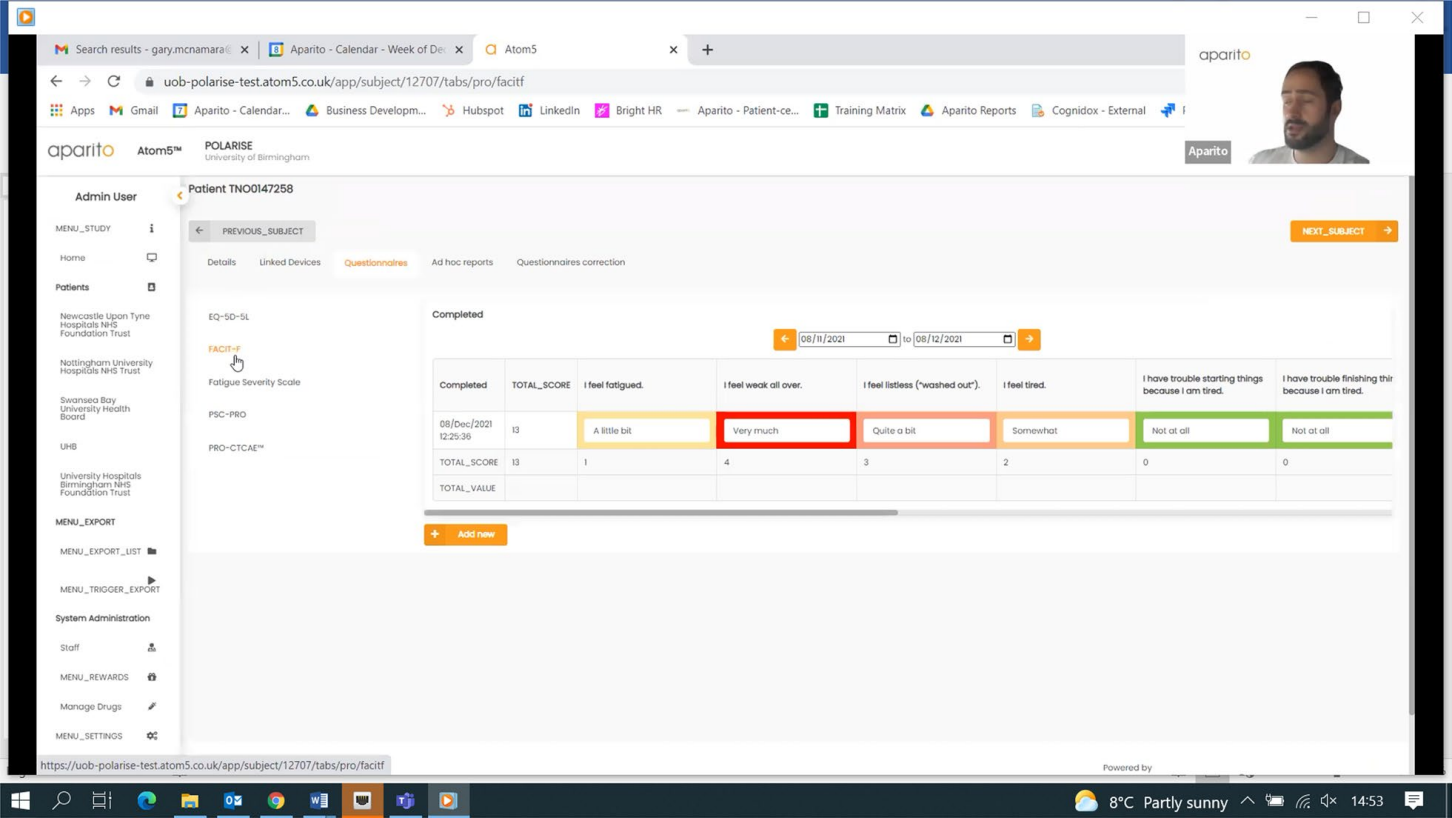
Screenshot of Atom5™ dashboard

**Fig. 3:**
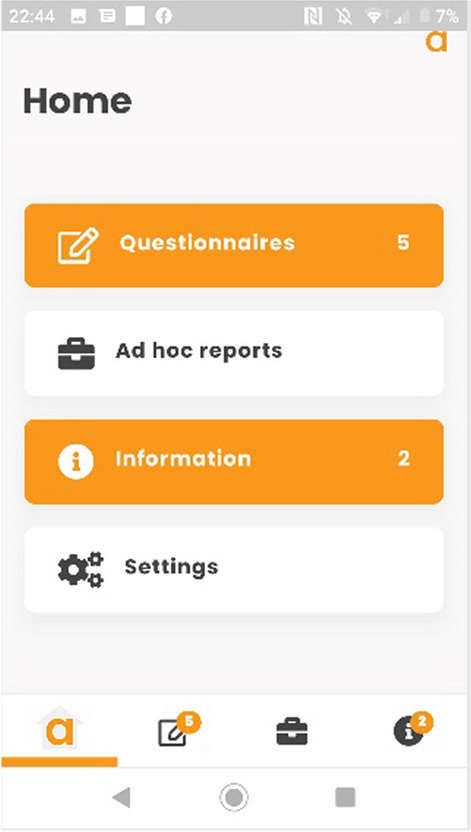
Screenshot of Atom5™ patient-facing app

**Fig. 4:**
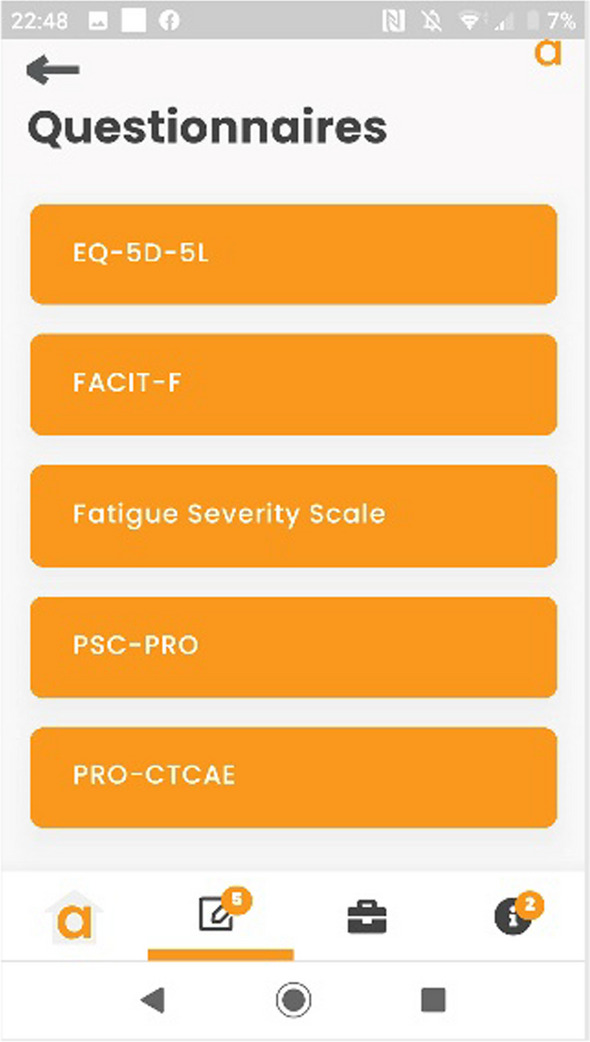
Screenshot of Atom5™ patient-facing app

The PROmics^R^ system is a configuration of Atom5™, developed by Aparito Ltd, a UK-based med-tech company. Study participants accessed a secure application (Atom5™) used by the study participants to complete the selected PRO measures on their own smartphone/tablet (iOS and Android). Atom5™ consists of two interfaces:

(i) A clinician dashboard accessed via a web browser onto which research team members (Research Nurses) input patient and their trial information as part of users’ registration; manage users’ information; and undertake data downloads (Figs. 1 and 2);
(ii) A patient-facing interface accessed via an app on Android/iOS devices onto which trial participants input their PRO data (Figs. 3, 4)

The development of the PROmics^R^ ePRO platform was informed by discussion with members of a local PPI group drawn from the original disease conditions. These PPI group members met monthly for 12 months to agree on the design and feature of the ePRO platform and contributed to the system requirement document Atom5™.[29, 30]

#### Selection of PROs measures

PSC, RA, LN, and CD are all associated with high symptom burden and reduced QoL. The advanced therapy product may reduce inflammatory processes resulting in an improvement or sustainment of symptoms and HRQoL. All PRO analyses were exploratory due to the small sample size of the trial and provided evidence to support later stages of the development programme. Alongside data on the safety and therapeutic effect of any given advanced therapy product, further evidence is required on its impact on patient symptoms, side effects and HRQoL, both immediately after delivery of the intervention and longer-term effects. As such, the rationale for PRO measures selection was based upon trial hypotheses that the use of the product would lead to:

- Improvement in fatigue and stabilisation of bowel-related symptoms for patients with PSC
- Improvement in joint pain, reduced disability and fatigue for patients with RA
- Improvement in fatigue and general well-being for patients with LN and CD

The findings of a systematic review of core outcome sets and recommended outcome measures across inflammatory diseases [31] were used to inform selection of PRO measures addressing key concepts of interest, in addition to psychometric properties, and patient preference [29].

Proposals for PROs were formulated and shared with the Trial Management and PPI Groups. These were reviewed in relation to trial objectives and by disease-specific patient and public involvement groups assessing alignment with patient preferences, research interests, and burden. Regulatory input was sought from the Medicines and Healthcare products Regulatory Agency (MHRA).

Outcomes measures were split between efficacy outcomes and tolerability. Efficacy outcomes measured the impact of the treatment on participants’ quality of life at pre-determined timepoints whereas tolerability outcomes measured toxicity more frequently and on an ad-hoc basis to have a better understanding of any tolerability issues in this early phase setting.

#### Patient-reported adverse events

As part of the POLARISE trial, existing PRO measures, item banks, and available patient-reported adverse event measures were reviewed by clinicians to be included alongside the existing trial adverse event (AE) infrastructure within the local clinical trials unit. When appropriate, recommended process for AEs were implemented within the POLARISE trial context.

#### Timepoints for assessment

Triallists and clinical leads were involved in selecting the timing of administration of PROs.

Timepoints for the assessment of PROs were selected based on: (1) the anticipated timescales for treatment effect; (2) PRO measure recall periods; and (3) the burden experienced by the participant and the length of time expected for its completion.

### Usability testing of the PROmics^R^ ePRO system

- Study participants and recruitment

Patients were recruited for usability testing from patient and public involvement (PPI) panels (the Liver and Gastro-Intestinal PPI Reference Group and the Birmingham Rheumatology Research Patient Partnership). After being sent the information sheets by the PPI groups leads, members of the research team (CM & SEH) contacted these participants to arrange a suitable date and time for the usability testing. Written informed consent was obtained from all the participants. In total, nine people with RA, PSC, LN or CD were recruited.

Seven Research Nurses were recruited from one trust. Similar to the PPI participants, CM and SEH contacted them to arrange a suitable date and time for the testing session after sending them information sheets and obtaining informed consent.

(b) Data collection

#### Testing sessions/Cognitive interviews

One-to-one testing sessions were held with research nurses and PPI participants. These sessions consisted of performing a series of specific tasks independently on the patient-facing app (PPI participants) or the clinician dashboard (Research Nurses) as well as answering debriefing questions afterwards. All testing sessions were held online and recorded via Zoom [32]. The tasks performed by the PPI participants and the Research Nurses are detailed in Box 1.

**Box 1:** List of tasks performed by PPI participants during testing sessions

PPI participants	
Downloading the app; Registering (onboarding) the app via a single-use QR code; Accessing, opening, completing and submitting the PROMs; Proceeding to the next questionnaire; Accessing the information section; Reviewing the training leaflet	
Research Nurses	
Accessing the clinician dashboard Creating a new user Selecting a disease cohort Generating a QR code Reviewing the training leaflet	

Cognitive interviews and “think aloud” techniques were used by the researchers (CM & SEH) during the testing sessions. These techniques aim to capture participants’ thought processes while performing specific tasks with the aim of gaining an understanding of how participants understand and interact with the ePRO system [33, 34]. After completing use of the system, participants were asked specific debriefing questions about their overall experience, any difficulties they had encountered as well as any other comments they had not shared with the researcher before.

The issues encountered by the PPI participants and the research nurses as well as the researchers’ notes and observations during the testing sessions were recorded (on video and in writing) to measure the effectiveness, efficiency and satisfaction of the patient-facing app and the clinician dashboard. All data collected was anonymised.

#### Satisfaction questionnaires/Demographic information

In addition to cognitive interviews, the researchers collected demographic information and assessed participants’ overall satisfaction with the ePRO system with satisfaction questionnaires. The bespoke satisfaction questionnaires consisted of four questions designed to rate satisfaction with the system and its usability on a 5-point scale (1—poor or no/5—excellent or yes).

(c) Data analysis

Comments from all the participants were recorded in a table which included general observations from the researcher, whether participants completed the tasks independently or with support, how they found the user experience, whether they understood the instructions and what could be done to improve the ePRO system (app and clinician dashboard). Two coders (CM & SEH) coded the comments and extracted themes. Any discrepancies between the two coders was settled by a third researcher (MC). Participant ratings for the four usability questions were used to calculate a mean score per question.

## Results

### Selection of PRO measures

The PRO aim was to gather (i) preliminary evidence of the impact of treatment on HRQoL, (ii) tolerability of treatment, and (iii) specific experience of treatment-related AEs. In addition, we aimed to assess feasibility of assessment to inform the design of future trials. The following PROMs were included in the PROmics^R^ study (Table 1): EQ-5D-5L, Fatigue Severity Scale, FACIT-Fatigue, and PSC-PRO. Two measures of fatigue were included as PPI members believed that each measure was aimed at different types of people and were unable to decide which one would be the most suitable to include.

**Table 1:** PROMs used in the PROmics^R^ study

PROM Category	PROM	Primary Sclerosing Cholangitis	Rheumatoid Arthritis	Lupus Nephritis	Crohn’s Disease	Rationale
GENERIC/ HEALTH ECONOMIC	EuroQol 5D-5L	8 timepoints		9 timepoints		Local health reimbursement requirement [58]
		The EQ-5D-5L has been widely validated and its use in resource assessments is specified by the National Institute for Health and Care Excellence (NICE) [59] It consists of five items and a visual analogue scale, it takes eight minutes to complete and has a same day recall period				
SYMPTOM SPECIFIC	Fatigue Severity Scale	8 timepoints		9 timepoints		Preferred by PSC patients, use in prior trials, methodological consistency and maximised comparability between arms [60]
		The FSS consists of nine items and requires less than five minutes to complete. It uses a two-week recall, and a 7-point Likert scale from 1 (strongly disagree) to 7 (strongly agree) A higher score indicates higher fatigue. It will be used in accordance with a user manual and is frequently used in a PSC population [61]				
	FACIT-Fatigue	8 timepoints		9 timepoints		Referenced by EMA and ICHOM for use in RA patients, and Methodological consistency and maximised comparability between arms [62]
		The FACIT-Fatigue is a validated measure [63] and consists of 13 items and takes five minutes to complete It uses a seven-day recall period and a 5-point Likert scale from 0 (not at all) to 4 (very much). Higher scores indicate less fatigue and better QoL				
DISEASE SPECIFIC	PSC-PRO	8 timepoints	–	–	–	Only PSC-specific PRO available and developed with extensive patient input [61]
		The PSC-PRO consists of 42 items and takes seven to fifteen minutes to complete It uses a 24-h recall period for the symptom module and a seven-day recall period for the impact module. A dichotomous/5-point Likert (1 Never—5 Always)/0–10 rating scale is used throughout. A high score indicates worse health status. It will be used in accordance with a user manual and has been developed and validated in a PSC population [64]				

### PRO-CTCAE™

The Common Terminology Criteria for Adverse Events (CTCAE) is routinely used in oncology as the means of categorising AEs in trials and is a descriptive terminology that evolved alongside other toxicity grading systems to become a comprehensive, multi-modality grading system [35, 36] for reporting the acute and late effects of cancer treatment. It can also be used to assess toxicity and side effects in non-oncology early phase trials. The PRO-CTCAE™ was developed to characterise the frequency, severity and interference of 78 symptomatic treatment toxicities that could be meaningfully reported from the patient perspective. It has been established that symptomatic adverse events are under-detected by clinicians versus patients, who are better at identifying low-grade symptomatic events [37, 38]. As such, the PRO-CTCAE™ could generate information that complements CTCAE data and can provide a more holistic insight into patient experience on treatment, which is integral for clinical decision-making. In oncology, the PRO-CTCAE™ has demonstrated favourable validity, reliability, and responsiveness [25]. However, the use of the PRO-CTCAE™ outside of oncology is novel [39]. The PRO-CTCAE™ was selected for use in the POLARISE trial to assess patient reported symptoms and potential side effects of therapy.

Due to the early-phase nature of the trial and limited availability of safety profile data, focused evidence identification was initially employed to identify a sub-selection of PRO-CTCAE™ items [40]. However, due to the limited AE reporting by previous studies, the use of different adjunct therapies across trials, and the interaction between underlying disease and physiology resulting in a different disease profile and a range of potential AEs, a scarcity of data was available. Therefore, we presented the entire item library to participants, along with a free text item. This approach addressed issues relating to the inconsistent AE evidence upon which to base item selection and potential channelling of patients to pre-selected items when using AE item sub-selection. This would also enable the development of an evidence base relating to the novel use of the PRO-CTCAE™ outside of oncology.

Choice of AE measures was undertaken in collaboration with the TMG, allowing for gaps in proposed AE measurement to be identified and addressed. Additional items (‘Feeling feverish’ and ‘Rectal bleeding’) were developed with the licence holders of the proposed PRO measures, with input from relevant clinical TMG members and PPI representatives in collaboration with the instrument developers.

Having identified patient-reported global tolerability as a necessary inclusion item [41, 42], the Functional Assessment of Cancer Therapy—General (FACT-G) [43, 44] was chosen as part of the AE and tolerability PRO battery. Specifically, the single item, FACT-G GP5 was used due to evidence of its validity for use outside of oncology and demonstration of its successful capture of data indicating treatment discontinuation [45].

Following discussion with clinical team patient partners, instrument developer and informal advice from regulatory colleagues, it was decided that a patient’s submission of a PRO-CTCAE™ and/or FACT-G GP5 report via the PROmics^R^ system with an absolute score of ≥ 3 and/or a PRO-CTCAE™ response of “yes” would initiate the auto-generation of single email per patient. The alert email is to be sent to relevant trial site Research Nurses and staff in the Cancer Trials Unit. Upon receipt of notification email, Research Nurses at site log on to the PROmics^R^ platform to view the data and validate. The data is assessed by the Research Nurse in accordance with disease group specifications and a CTCAE assessment will be undertaken as required. The assessment is recorded and retained in the patient notes and added to the CRCTU database as per protocol.

The final battery of PROMs was reviewed and agreed by the Trial Management Group and patient partners. Initial completion is estimated can be undertaken in < 30 min and subsequent entries in < 20 min. The time taken to complete the PROs electronically is recorded through use of time-stamps for data entered via the PROmics^R^ study app. The follow-up period is 24 months.

### Timepoints for assessment

PRO-CTCAE™ and FACT-G GP5 are collected on a weekly basis, in clinic or at home with ad hoc reporting available for participants if symptoms change. The remaining PROs are collected at pre-specified timepoints over the 24-month follow-up period, depending on the disease cohort. Further details are provided in the study protocol schedule of events.

### Cognitive interviews

Nine PPI participants with PSC, RA, LN or CD and seven Research Nurses took part in a testing session of the PROmics^R^ ePRO system. Table 2 presents the PPI members’ characteristics. All participants but one reported being comfortable using mobile technology. The testing sessions lasted between 12 and 55 min.

**Table 2:** PPI group members’ characteristics

Variable	N
Age	
< 55 years	3
> 55 years	6
Gender	
Male	4
Female	5
Ethnic group	
White	5
Asian/Asian British	1
Black/African/Caribbean/Black British	2
Mixed/multiple ethnic groups	1
Disease group*	
Rheumatoid arthritis	5
Primary Sclerosing Cholangitis	1
Lupus Nephritis	1
Crohn’s Disease	1
Other	3

*several participants selected more than one option

Five participants used an iOS device and three used an Android device (one was not recorded).

### PPI participants

#### Effectiveness of patient-facing ePRO app

Seven of nine participants were able to complete the testing session; two participants failed at outset due to an outdated operating system on their device (n = 1), and a compatibility issue which prevented registration (n = 1). Of the seven who were able to register, issues were identified ranging from technical issues (such as some radio buttons not working, or missing information resources) to human factors (difficulties navigating the layout) (Additional file 1: Table S1). Participants believed that the length of the PROCTCAE™ was appropriate in order to understand any tolerability issues. In addition, although some patients felt that the PROCTCAE™ questionnaire was lengthy at first, they also recognised that, depending on their symptoms, they would not have to answer each question. Further feedback will be obtained during the ongoing feasibility study.

#### Satisfaction with the patient-facing ePRO app

Patient participants’ satisfaction was high amongst those who were able to complete testing. These 7 participants scored it highly on ease of use, content and visual display, with all participants stating that they would be ‘likely’ or ‘very likely’ to recommend it to others (Additional file 1: Table S2). When asked to reflect on their use of the system, these participants highlighted its strengths including its simplicity and responsiveness, whilst also stating what they did not like (e.g. length of questionnaire, the use of the VAS scale) and feedback to improve its usability (e.g. insertion of a progress bar). One participant noted that ‘Seems like a good replacement to paper PROs (Participant 3)’.

The researchers noted that although the participants liked the design, layout, and colour of the training leaflet, they did not always use it during the testing sessions, preferring to consult the researchers. Participants found it challenging to simultaneously use Zoom, refer to the training manual, navigate, and complete the PROMs on the app.

### Research nurses

#### Effectiveness of the clinician dashboard

Overall, the Research Nurses found the process of navigating through the clinician dashboard and completing the tasks straightforward. They encountered few issues during the process. These issues are summarised in Additional file 1: Table S3. They included not being able to log into the dashboard and not being able to create a new participant because of a duplicate participant number. After the researcher advised the research nurses to use a different internet browser and a different participant number, they were able to continue with the testing session.

During the testing sessions, the research nurses commented on other aspects of the clinician dashboard not included in the tasks, such as the presence of coloured boxes. As they were reflecting on their potential meaning, they suggested that these colours could be used to identify those participants who needed attention depending on potential alerts triggered by their answers to the PROMs. Similar to the PPI participants, CM and SEH noted that the Research Nurses did not all refer to the training manual when they were unsure how to complete a task.

### Impact on the ePRO system

The usability testing sessions conducted with PPI participants and research nurses highlighted a number of minor issues that were then reported to the ePRO system developers in order to improve it and make it more accessible to users ready for deployment in the clinical trial.

## Discussion

This article presents the results of the development and usability testing of an ePRO system developed for use by participants with inflammatory conditions and Research Nurses in a phase II ATMP basket trial. Our ePRO system was informed by existing literature, regulatory guidance, patients, and clinicians. Four PRO measures, along with PRO-CTCAE™ and a global tolerability question were selected to be programmed onto ePRO system. The results of the cognitive interviews suggest that the PROmics^R^ ePRO system was a suitable way to collect PROs in the POLARISE trial. Both the PPI participants and the research nurses found the app and clinician dashboard respectively easy to use, despite a couple of participants encountering a technical issue.

Assessment of PROs in clinical trials provides valuable insights into the patient perspective and enables participants’ disclosure of severe or worsening symptoms that may require immediate clinical attention. Use of formal PRO alert systems in safety monitoring enable structured and planned responses to these data that promote patient safety while avoiding potential bias [46, 47]. Linking patient reports with symptom monitoring systems has been found to be associated with clinical benefits such as better health-related quality of life and less use of emergency medical services and hospitalisation [20].

While ePROs are an important step forward towards patient-centred care, we need to recognise the risks that are involved with moving from PROMs to ePROMs. In this paper, we have carefully documented this process in order to share the learning that may be applicable to others developing ePROs. The risks include privacy issues [48], technical difficulties [49], large financial investments [50], and issues around diversity, equity and digital exclusion [51, 52]. The latter is one of the most prominent risks and it refers to failing to reach people, either because they do not have access to digital technology (mobile phone/tablet/Internet) or because they do not know how to use it [48, 53]. The findings of our study showed that although all our participants had access to their ‘Bring Your Own Device’ (BYOD), one participant had an incompatible version of iOS and was not able to complete the testing. In addition, another one admitted not being technology savvy and asked their partner to help him download the app and navigate through it. The age (> 55) could perhaps explain the low level of digital literacy, however, this was not necessarily reflected in the rest of the other over 55 PPI participants in our study and other studies [22, 54]. Recent research has shown that using smartphones makes digital communication more accessible to people with lower levels of digital literacy [55]. The support given to this participant by their partner during the testing session raises the issue of how much influence this external support has on the participant’s answers. We therefore recommend ensuring that participants’ devices are compatible with the ePRO system and providing an alternative solution to address digital poverty as well as ensuring inclusion, such as the provision of a device for the duration of the trial or paper-based PROMs, even if using paper-based PROMs might affect the overall reporting of PROs. Other options might be to ensure that the ePRO system is designed to be used with Smartphones and tablets or with older iterations of operating systems or to provide participants with a device, thereby not expecting them to use their own devices. Even amongst those who are not excluded, usability issues including length or content and delays in loading may lead to ePRO fatigue, which causes failure to complete and disengagement of some participants over time. Digital exclusion and digital disengagement should be taken seriously. They can be safety issues (for example if they form part of the safety monitoring of the study through notifications/alerts to the clinical team), but also can introduce significant bias, since not all parts of the target population will be represented.

It was not possible to discern alerts from usability testing as patients had not received cell therapy. This will be evaluated as part of the feasibility which is currently being conducted [28]. Patients’ views on using the ePRO system will also be collected using qualitative methods as part of the ongoing feasibility study.

### Implications for ePRO developers

Some of the participants mentioned issues with scrolling down the page to click on a ‘Submit’ button in order to submit the questionnaire and move to the next page. It is possible that the fact participants had to scroll down to see and click on the ‘Submit’ button was due to the fact that they were using a smartphone or iPhone. Although there is a definite advantage to using a portable device to report symptoms, programmers need to ensure its use is as simple and intuitive as possible for participants. This is even more true in the case of our PPI participants, some of whom have a diagnosis of RA and consequently experience difficulties with manual dexterity. Although none of them seemed to be affected by the scrolling issue, it is something that is worth bearing in mind for other people with a similar condition who might be affected by it [56]. People affected by this could use a stylus to help them scrolling through the questions.

The fact that the PPI participants and the Research Nurses did not refer to all refer to the training manual while testing the ePRO system poses the question of its usefulness. One of the explanations for not using the manual could be because the two researchers, CM and SEH, were present and it was easier for the participants to ask questions directly to the researchers. Looking for the answer to their question in the manual would have been more time-consuming. Developers could perhaps include prompts on the screen, which might offer support and be more timesaving.

In addition, developers need to leverage accessibility options available as standard within smartphones and tablets, such as change text size or touch controls to ensure inclusivity and maximise participation.

### Strengths and limitations

The main strength of our study is that the development of an ePRO system that brings together multi-sector, multi-disciplinary expertise in PROs, app development, medical regulation, and clinical expertise. In addition, the usability testing phase includes the experiences of PPI participants and of research nurses.

The methodology used which includes cognitive debriefing methods, such as “think aloud” techniques provide insights into real-time feedback and emotional responses involved in using the ePRO system which is not always accessible with more traditional qualitative data collection methods [57].

Finally, our study builds on a previously validated PROM (PRO-CTCAE™) and provides useful information on feasibility of assessment but only preliminary efficacy/tolerability data.

We recognise that our study has some limitations. Whilst we actively sought to sample a diverse range of users, the small sample size has limited the diversity within the user group. This means that our sample might not be representative of the whole population. One of the reasons for this is that participants were selected from Patient and Public (PPI) groups. Although participants could have been recruited from NHS sites, it would have meant obtaining NHS ethical approval and would have increased the overall length of the study, which we could not afford to do. This will be addressed in the next stage of testing and development as part of the POLARISE trial, along with other features of the PROmics ePRO platform, such as administration burden, reporting of PRO alert data, and clinical interventions based on PRO data [28]. Due to COVID-19 restrictions, all assessments had to be done virtually. This meant that all participants had to use new technology (ePRO app and Zoom) and the printed training guide concurrently. Some participants did not refer to the training manual, preferring to ask the interviewer for clarification as they progressed through the ePRO system. Although we recorded the time participants took to complete each task, this data was not included in the analysis. Indeed, as participants stopped to ask questions whilst completing each task, we felt that the recorded time did not accurately reflect the reality and made it difficult to assess the efficiency of the ePRO system. Finally, although this study identified reasons why a motivated person might be unable to use an ePRO system (e.g. device incompatibility), this study did not explicitly look at other aspects of digital exclusion in the context of ePROs such as those who would not wish to engage with a digital process, or who do not have the physical or mental faculties to reliably use it.

## Conclusion

Developing an ePRO system with the input from existing literature, regulatory guidance, patients, and clinicians and testing its usability testing are important steps in its design and implementation. By testing the effectiveness, efficiency, and satisfaction of our novel ePRO system (PROmics^R^), we learnt that most people with an inflammatory condition found it easy to report their symptoms using an app on their own device. Their experiences using the PROmics^R^ ePRO system within a trial environment will be further explored in our upcoming feasibility testing. Research nurses were also positive and found the clinical dashboard easy-to-use. Using ePROs in early phase trials is important in order to provide evidence of therapeutic responses and tolerability, increase the evidence based, and inform methodology development.

### Supplementary Information

**Additional file 1.** Suppplementary tables 1–3.

## Data Availability

The datasets used and/or analysed during the current study are available from the corresponding author on reasonable request.
