# Supplementary material for: Development and usability testing of an electronic patient-reported outcome (ePRO) solution for patients with inflammatory diseases in an Advanced Therapy Medicinal Product (ATMP) basket trial
Source: J Patient Rep Outcomes. 2023 Oct 9;7:98. doi: 10.1186/s41687-023-00634-3 (PMC10562321; doi:10.1186/s41687-023-00634-3)
Supplement: Supplementary file 1 — Additional file 1. Suppplementary tables 1–3. [file 41687_2023_634_MOESM1_ESM.docx]

**SUPPLEMENTARY TABLE 1**

**Supp. Table 1 – Issues encountered by participants during testing sessions**

| **Tasks** | **Issues encountered during the testing session** |
| --- | --- |
| Downloading app | Wrong iOS version* (n=1)  Difficulty finding app on app store (n=1) as app logo in app store/Play Store did not match what was in training leaflet |
| Onboarding/ Registering  (QR code) | Type error – cannot read property ‘Garmin’ of undefined* (n=1) |
| Accessing PROMs | PROMs were slow to appear on app (n=5) |
| Opening PROMs | None |
| Completing PROMs | Issues with app design & programming functions  All PROMs:  -length of time it took for PROMs to load  EQ5D:  -Issues with scrolling to “Next” button  PRO-CTCAE^TM^:  -Sometimes confused by layout and question logic (clicking on a symptom opens up more questions)  PSC-PRO/PRO-CTCAE^TM^:  -Some of the functions not working (radio buttons/VAS) |
| Submitting PROMs | PROMs still appearing in list after submitting (app) (n=2) |
| Proceeding to next Questionnaire | None |
| Training leaflet | Information on which version of Android/iOS work with ePRO system is missing |

*issues which led to the testing session being abandoned as the issue could not be resolved and the system could not be used.

**SUPPLEMENTARY TABLE 2**

**Supp. Table 2 – Satisfaction with ePRO system**

| **Question** | **Average score (5-point scale) (range)** |
| --- | --- |
| Ease of use and navigation | 4.4 (4.0-5.0) |
| Satisfaction with content | 4.4 (4.0-5.0) |
| Satisfaction with visual display | 4.5 (4.0-5.0) |
| Likelihood of using again or recommending to others | 4.5 (4.0-5.0) |

**SUPPLEMENTARY TABLE 3**

**Supp. Table 3 – Issues encountered by research nurses during testing session**

| **Tasks** | **Issues encountered during the testing session** |
| --- | --- |
| Access to clinical dashboard | Not able to log in (on Firefox) – this was fixed when the research nurse switched to Chrome (n = 1) |
| Creating a new user | The order of the tasks did not match the training guide (all participants)  “Error – Duplicate subject number” (n = 1) |
| Selecting a disease cohort | None |
| Generating a QR code | Unsure of where to click (n = 1) |
| Review training leaflet | Some instructions were missing |
